# A mathematical model of mitochondrial swelling

**DOI:** 10.1186/1756-0500-3-67

**Published:** 2010-03-11

**Authors:** Sabine Eisenhofer, Ferenc Toókos, Burkhard A Hense, Sabine Schulz, Frank Filbir, Hans Zischka

**Affiliations:** 1 Institute of Biomathematics and Biometry, Helmholtz Zentrum München, German Research Center for Environmental Health, Ingolstädter Landstrasse 1, 85764 Neuherberg, Germany; 2 Mathematics Centre of the Technischen Universität München, Boltzmannstrasse 3, 85748 Garching, Germany; 3 Institute of Toxicology, Helmholtz Zentrum München, German Research Center for Environmental Health, Ingolstädter Landstrasse 1, 85764 Neuherberg, Germany

## Abstract

**Background:**

The *permeabilization *of mitochondrial membranes is a decisive event in apoptosis or necrosis culminating in cell death. One fundamental mechanism by which such permeabilization events occur is the calcium-induced mitochondrial permeability transition. Upon Ca^2+^-uptake into mitochondria an increase in inner membrane permeability occurs by a yet unclear mechanism. This leads to a net water influx in the mitochondrial matrix, mitochondrial swelling, and finally the rupture of the outer membrane. Although already described more than thirty years ago, many unsolved questions surround this important biological phenomenon. Importantly, theoretical modeling of the mitochondrial permeability transition has only started recently and the existing mathematical models fail to characterize the swelling process throughout the whole time range.

**Results:**

We propose here a new mathematical approach to the mitochondrial permeability transition introducing a specific delay equation and resulting in an optimized representation of mitochondrial swelling. Our new model is in accordance with the experimentally determined course of volume increase throughout the whole swelling process, including its initial lag phase as well as its termination. From this new model biological consequences can be deduced, such as the confirmation of a positive feedback of mitochondrial swelling which linearly depends on the Ca^2+^-concentration, or a negative exponential dependence of the average swelling time on the Ca^2+^-concentration. Finally, our model can show an initial shrinking phase of mitochondria, which is often observed experimentally before the actual swelling starts.

**Conclusions:**

We present a model of the mitochondrial swelling kinetics. This model may be adapted and extended to diverse other inducing/inhibiting conditions or to mitochondria from other biological sources and thus may benefit a better understanding of the mitochondrial permeability transition.

## Background

In 1976 Hunter, Haworth and Southard described a calcium induced non-specific increase in the permeability of the mitochondrial inner membrane of isolated beef heart mitochondria, which they termed *mitochondrial permeability transition *(MPT) [1]. Such a permeability transition is lethal because it results in the release of death inducing molecules from and/or in metabolic failure of mitochondria [2]. Since then a plethora of studies have dealt with this phenomenon studying the mechanistic, molecular and pharmacological aspects of the MPT [3]. Major findings were the discovery that the MPT could be inhibited by submicromolar concentrations of the immunosuppressant drug cyclosporine A (Cys A) [4] and the electrophysiological characterization of the MPT as mediated by a pore/channel-like structure [5,6]. As had been realized early on, the increased permeability of solutes towards the matrix space causes an osmotically driven water influx [1]. The concomitant extension of the mitochondrial inner membrane ("swelling") leads to a matrix transition from the aggregated to the orthodox state [1], culminating in the permeabilization and rupture of the outer mitochondrial membrane [7-9]. Despite these findings important issues concerning the MPT have remained unanswered or controversial. It is for instance a matter of continuous debate which components exactly build up the MPT pores [3,10-12].

To improve the understanding of the kinetics and complex interdependences of the MPT process, modeling of the MPT pore function has only started recently with two conceptually different approaches.

One is mainly oriented on a detailed biochemical and biophysical description of mitochondrial molecular processes such as the mitochondrial respiration, ion exchanges, the mitochondrial transmembrane potential, etc. [13,14]. For each of these processes an equation is created which are combined in a system of nonlinear ordinary differential equations including a number of variables (e.g. the amount of Ca^2+ ^in the mitochondrial matrix, pH-value, or the inner transmembrane potential). The specific advantage of this approach is that it can reproduce the three different states of the pores: closed, open in the low-conductance mode, or open in high-conductance mode. However, this model does not display the time course of pore opening and lacks a major feature, the irreversible swelling upon volume increase, and thus it is inadequate for simulating mitochondrial swelling.

**Figure 1:**
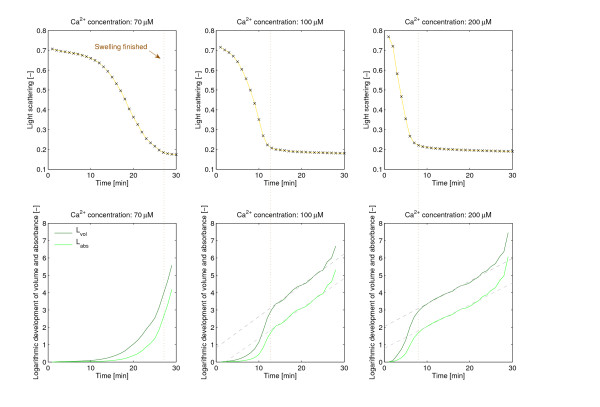
**Swelling curves and the development of the logarithmic volume and absorbance**. , where *V*_0 _is the initial volume before swelling, *V*_*p *_the final volume after swelling, and *V *(*t*) the volume at time *t*. Similarly, , where *A*_0 _is the initial, *A*_*p *_the final absorbance, and *A*(*t*) the absorbance at time *t*. Both *L*_*vol*_(*t*) and *L*_*abs*_(*t*) were obtained from the experimental data. The vertical dotted lines show the end of the swelling, the slanted dashed lines are the best linear approximations to the curves.

The other modeling approach attempts to directly represent mitochondrial swelling. It focuses on the basic kinetic process, thus it is mathematically and numerically comparatively easier to handle. It has only one [15] or two [16] equations and concentrates on the rate of increase of the number of swollen mitochondria, largely ignoring the details of the underlying biochemical processes. Despite the simplifications this approach can produce a more accurate picture of the uptake of calcium in the mitochondrial matrix, which can be directly compared with the experimental data. To our knowledge, Massari [15] created the first model of this kind assuming first order kinetics. A great advantage of this model is that it can be solved explicitly due to its lower mathematical complexity in contrast to the above mentioned approaches. A drawback of this model - as partly mentioned already in [15] and later pointed out also in [16] - is that it fails to account for the initial lag phase in mitochondrial swelling. In agreement with these reports we have observed that the Massari model especially fits the end, the "tail" of the swelling curves, but misses their starting phases. The reason for this is the assumption in the derivation of the model, according to which the logarithm of the *volume *of mitochondria (*L*(*t*)) changes linearly during the swelling process. In fact, this linearity only occurs once most of the actual swelling is done (see Figure 1). Recently Baranov *et al*. [16] presented an elaborate model, which provides a good simulation of the swelling on a longer time scale. It consists of two differential equations - one for the amount of calcium and one for the ratio of swollen mitochondria. The authors take into consideration that the Ca^2+^-uptake by mitochondria occurs in several steps with different reaction rates. However, their simulations concentrate on the middle part of the experimental swelling curves, and unfortunately do not explain the above mentioned "tail". The change of the parameters is tested and discussed in dependence of various inducers and inhibitors, but the variation of parameter values with increasing amount of Ca^2+^-addition was not examined.

Experimental studies of the swelling process often use populations of isolated mitochondria, in which the swelling process is initiated by the addition of inducing substances, naturally and in our experiments Ca^2+^. Quantitative analysis of the kinetic parameters of this process enables a deeper understanding of the underlying processes, a comparison between different mitochondrial populations, or renders information about the effect of potential drugs. Thus, it is of importance to develop a mathematical model, based on the known major functional properties of the process, which allows to analyse these parameter values. Reconciling the model with experimental data during the development process allows model optimization, and by this also helps in understanding the overall process.

The aim of our study is to develop a mathematical model allowing to analyse the kinetics of the complete swelling process. Based on experimental data, we assume that three subpopulations of mitochondria with different volumes exist: non-swollen, swelling and mitochondria that have completely finished the swelling. The first and the last group have constant mean volumes, depending only on their source and the medium. The mean volume of swelling mitochondria additionally depends on the characteristics of the swelling process, which could be influenced e.g. by properties and concentrations of added substances. The onset and the time course of the swelling typically varies between the mitochondria of different sources [17-19], caused e.g. by different sensitivity to inducers.

The dynamical behaviour of the total volume of the mitochondrial population, i.e. the integrated volume of all subpopulations of mitochondria, are analysed experimentally by their light scattering behaviour.

Swelling inducers (or inhibitors) directly or indirectly act on the PTP. Transfer of the externally applied substances into and accumulation within the mitochondria as well as the evolution of their possibly multistep activity are main causes of the delay phase, typically found between application and the start of the swelling process. As an example, Ca^2+^is transferred via the calcium uniporter into the mitochondria [10], and induces mitochondrial swelling via the binding to an opening-inducing Ca^2+^-binding site on the matrix side of mitochondria [10].

Our model is based on the above described subpopulation behaviour and delay, and consists of one differential equation which can be explicitly solved and one additional delay equation. After showing that our model is able to explain the experimentally found swelling dynamics for mitochondria from rat livers, parameter values were estimated. We found a positive feedback in the swelling process mediated by Ca^2+^. Our model is also capable of explaining the initial, short-term shrinking of mitochondria preceding the actual swelling as was documented for example in [20,7] and [17].

Taken together, this new model provides an optimized characterization of the swelling process and constitutes a base for the future study of the MPT, e.g. in mitochondria from other organs or species. As mentioned above, mitochondria from different organs (liver, heart, brain) have different sensitivity to permeability inducers and hence different swelling behavior. With the help of our model these differences can be quantified and better understood. Furthermore, although we have focused on Ca^2+^-induced swelling here, the model does not explicitely refer to the Ca^2+^-concentration, thus this model is also applicable to other inducers of MPT pore opening, like heavy metals, peroxides etc., or for the study of effects of diverse MPT-inhibitors. We present preliminary results comparing the swelling of liver and kidney mitochondria, as well as the comparison of Ca^2+ ^and Hg^2+^-induced swelling in liver mitochondria.

## Methods

### Experimental procedures

Mitochondria were isolated from rat livers of the LPP rat strain (ATP7B (+/-), [21]). Animals were housed in plastic cages with wooden fiber bedding (Lignocel-fiber (Rettenmaier&Söhne, Rosenberg, Germany). The animals were maintained in a room at 25°C and 30 - 40% humidity on a 12 h light/dark cycle. Water and Altromin 1314 diet (Altromin, Lage, Germany) was given ad libitum. Animals were killed under ether anesthesia by exsanguination through the vena cava and the liver was flushed with icecold saline through the portal vein. All animals were treated under the guidelines for the care and use of laboratory animals of the Helmholtz Zentrum München according to the FELASA (Federation of the European Laboratory Animal Science Associations).

#### Isolation of rat liver mitochondria

Mitochondria were isolated by *differential centrifugation *according to standard protocols. Briefly, freshly removed liver tissue was cut into pieces and homogenized with a glass teflon homogenizer in isolation buffer (5 mM TES, 300 mM sucrose, 0.2 mM EGTA, pH 7.2 with KOH). Homogenates were cleared from debris and nuclei by two times centrifugation at 800 g (10 min at 4°C). Mitochondria were pelleted from the supernatants at 9000 g (10 min at 4°C), washed two times at 9000 g (10 min, 4°C) and resuspended in isolation buffer.

#### MPT measurements of isolated rat liver mitochondria

MPT-induced osmotic swelling of freshly isolated rat liver mitochondria suspensions upon Ca^2+^-addition was routinely measured by light scattering at 540 nm in a micro-plate absorbance reader (*μ*-Quant™, Bio-Tek, Bad Friedrichshall, Germany) over a period of 60 min at RT. The final assay volume was 200 *μ*l, containing mitochondria at 0.5 mg/ml in "standard swelling buffer" (10 mM MOPS-Tris pH 7.4, 200 mM sucrose, 5 mM succinate, 1 mM P_*i*_, 10 *μ*M EGTA and 2 *μ*M rotenone). CsA (5 *μ*M) was added 4 min before Ca^2+^. Alamethicin as MPT-inducing agent was used at 1 *μ*g/mg mitochondrial protein. Intactness of the mitochondrial preparations was routinely checked by standard respiratory measurements.

#### Optical density measurements of swollen and unswollen mitochondria

We tested for a linear relationship between optical density readings and altering ratios of swollen, i.e. calcium treated mitochondria and control mitochondria. Thereto isolated rat liver mitochondria were treated with high dose calcium (300 *μ*M) to ensure complete swelling [7]. Subsequently treated and untreated control mitochondria were purified by *free flow electrophoresis *as described in [22]. Purified control mitochondria and Ca^2+^-swollen mitochondria (termed M2 mitochondria in [22]), were mixed at different ratios and optical readings performed at 260 nm.

### Computational methods

For the computations we used Matlab (The Mathworks^®^). The simulated volume curves were linearly rescaled to the optical density measurements and then the parameter values were optimized with least squares using the Nelder-Mead simplex method [23].

## Results

In this study we aimed at the development of a mathematically straightforward model which describes the mitochondrial swelling process quantitatively.

### Mathematical model

The model is based on the observation that mitochondria vary concerning their sensitivity for swelling induction by stimuli as Ca^2+ ^[22]. We model the time progress of the swelling with two equations for two variables. Let *X*(*t*) denote the fraction of mitochondria that is swollen or has started swelling at time *t *(0 ≤ *X*(*t*) ≤ 1) and *V *(*t*) the average volume of mitochondria at time *t*. Obviously *X*(0) = 0. Let *X*_*p *_be the ratio of swollen mitochondria after the whole swelling process is done (*X*_*p *_< 1). Let *V*_0 _and *V*_*p *_denote the volume of unswollen and totally swollen mitochondria, respectively.

The permeability transition process can be described via the initial value problem(1)

where *a *and *b *are parameters, in accordance with the experimental data. This form allows first and second order kinetics as well, depending on whether *a *= 0, and proved to be general enough. We also tested for the possibility of different orders, replacing the first factor on the right-hand side by *a*(*X*(*t*))^*r *^+ *b *and leaving *r *as a parameter, but the optimal value for *r *turned out to be always close to 1 (between 0.6 and 1.6), and the error term in the fitting to the data was only insignificantly better than the one obtained by assuming *r *= 1.

It can be seen with the separation of variables that the explicit solution of (1) is:(3)

Equation (3) shows that *X*(*t*) is monotone increasing with one inflection point (changing from convex to concave) and that lim . The solution is also robust in the sense that the rate of convergence is exponential.

We split up the mitochondrial volume *V *(*t*) into three different subpopulations, *V*_1_, *V*_2 _and *V*_3_. With the delay term *τ *denoting the average swelling time of a single mitochondrion, we have

By taking(4)

we obtain(5)

The parameter *k *determines the average volume of the mitochondria which are swelling. Naturally, *k *cannot be arbitrarily close to 0, it has a positive lower bound depending on the experimental setting (type of mitochondria, Ca^2+^-concentration, inhibitors, etc.). Setting the average volume to *kV*_*p *_necessarily leads to a small break of the curve at *t *= *τ*. This results from the fact that the right-hand side derivative of *X*(*t*) at *t *= 0 is lim_*t*→0+ _*X'*(*t*) = *bX*_*p *_and hence lim_*t*→*τ*+ _*V'*(*t*) *- *lim_*t*→0*τ*-_*V'*(*t*) = *bX*_*p*_*V*_*p*_(1 - *k*). Nevertheless, the size of the break tends to 0 as *b → *0. As most of our experiments showed rather low and almost invariant values for *b*, we could largely eliminate the break. Instead of considering only 3 discrete sub-populations of mitochondria, describing the fraction of swelling mitochondria by a term which integrates the volumes of a population of continuously swelling mitochondria over time could avoid the break, but this solution turned out to be hardly numerically treatable, hence we worked with the above defined *V*_1_, *V*_2 _and *V*_3_.

*X*_*p*_, *V*_0 _and *V*_*p *_are constants in the model, *a*, *b*, *k *and *τ *are the parameters.

### Modeling results

#### Parameter estimation by the experimental data

**Figure 2:**
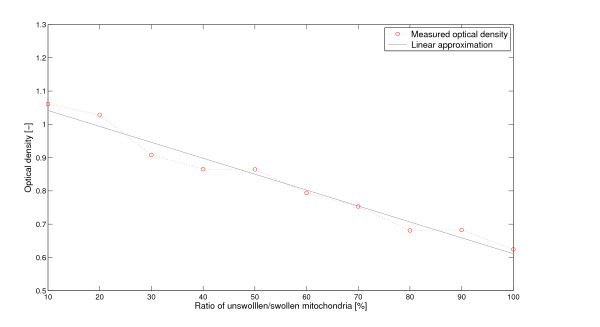
**Optical density measurements for different concentrations of swollen - unswollen mitochondria**. Isolated rat liver mitochondria were treated with high dose calcium (300 *μ*M) to ensure complete swelling [7]. Subsequently treated and untreated control mitochondria were purified by *free flow electrophoresis *as described in [22]. Purified control mitochondria and Ca^2+^-swollen mitochondria (termed M2 mitochondria in [22]), were mixed at different ratios and optical readings performed at 260 nm. The numbers below the columns show the ratio of unswollen/swollen mitochondria in percentages.

A prerequisite to apply the volume equation to the light scattering measurements is the knowledge of the relation between the optical density and the fraction of swollen mitochondria. In agreement with findings reported by Baranov *et al*. [[16], p. 667, Figure 1] and Petronilli *et al*. [[7], p. 21940, Figure 2] we have determined this relation to be linear (see Figure 2).

**Figure 3:**
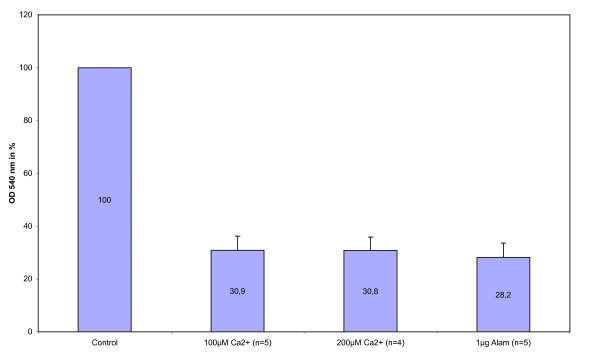
**Comparison of light scattering values of swollen mitochondria induced by Ca^2+ ^and alamethicin**. Relative optical density values at 540 nm of rat liver mitochondrial suspensions upon swelling induction by calcium or alamethicin after 60 min. OD values of CsA/Ca^2+^-treated (i.e. swelling inhibited) mitochondria after 60 min served as control and were set to 100%; n is the number of independent biological repeats.

The difference in optical density observed between the untreated and the fully swollen mitochondria compared to the decrease of optical density during the Ca^2+^-induced swelling leads to the conclusion that only a relatively *low percentage of mitochondria did not swell*. That is why we chose the estimated value *X*_*p *_= 0.9, which is higher than the values used in [15]. Massari *et al*. have reported incomplete mitochondrial swelling upon calcium induction and therefore determined the value of *X*_*p *_by adding alamethicin after the Ca^2+^. Their rationale was that alamethicin acts as a pore forming agent [24] and is thus assumed to cause maximal swelling due to a direct induction of a harsh osmotic imbalance between all mitochondria and their surrounding media. In contrast to these observations, we did not find a difference in the extent of mitochondrial swelling induced by either alamethicin (at 1 *μ*g/mg mitochondrial protein) or calcium at higher concentrations (see Figure 3). A possible reason for this discrepancy may be a shorter observation time of the swelling process in the study of Massari *et al*. indicating that the calcium-dependent swelling was not complete. The other two constants were set as *V*_0 _= 1.2 ml/mg protein and *V*_*p *_= 1.7 ml/mg protein [14].

It is worth mentioning that the chosen values of the constants *X*_*p*_, *V*_0_, and *V*_*p *_do not significantly modify the outcome of the simulations, because of the linear rescaling to the optical density measurements as mentioned in the Methods section. Thus, a change of these values would change neither the shape of the volume nor that of the simulated swelling curves. Furthermore, although optimal values for these parameters may differ slightly from the chosen ones, their mathematical dependence (e.g. exponential/linear) would not change.

#### Simulated swelling curves

**Figure 4:**
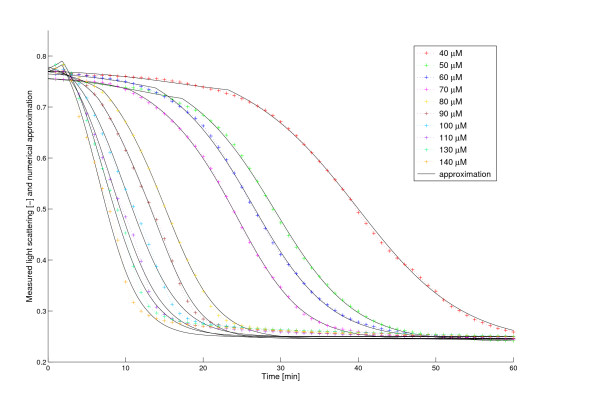
**Simulated swelling curves and experimental data**. The color lines with the + signs are the swelling curves from the experiments at different Ca^2+^-concentrations (40 - 140 *μ*M). The solid black lines are the corresponding rescaled numerical approximations of the volume (*V *(*t*)) obtained from the model. *V *(*t*) was rescaled linearly (cf. Methods - Optical density measurements) considering the highest and lowest values of the measured light scattering data and the corresponding simulated volume curve, respectively.

Figure 4 shows the experimental data and the rescaled simulated volume curves for various Ca^2+^-concentrations and Table 1 shows the optimal parameter values. We found an accurate fit of the volume curves and the measurements. As the simulation assumes a second order behaviour, a positive feedback loop exists within the system.

**Table 1:** Optimized model parameters with fixed *b *≡ 0.021.

Ca^2+^	*τ*	a	k	err	Δ *err*
40 *μ*M	25.7510	0.1174	0.7341	1.7233e-03	+1.01e-03
50 *μ*M	18.0899	0.1882	0.7370	8.5859e-04	+1.31e-04
60 *μ*M	15.4921	0.1965	0.7362	7.6069e-04	+1.35e-04
70 *μ*M	13.7992	0.2257	0.7442	1.3933e-03	+7.30e-04
80 *μ*M	7.1833	0.3194	0.7657	2.5434e-03	+8.77e-07
90 *μ*M	6.5893	0.3734	0.7971	3.7028e-03	+5.38e-05
100 *μ*M	2.2030	0.3182	0.7831	4.5558e-03	+5.27e-06
110 *μ*M	2.0000	0.3660	0.8596	5.4100e-03	+3.20e-04
120 *μ*M	2.0000	0.3513	0.8854	5.7481e-03	+4.35e-04
130 *μ*M	1.9999	0.3967	0.9300	6.9423e-03	+5.51e-04
140 *μ*M	1.8418	0.4554	0.9694	8.5506e-03	+7.98e-04

The Table shows the optimal parameter values in case we keep the value of *b *fixed. *err *is the least squares error, Δ *err *shows the difference in the least-squares error between the models with fixed and variable *b*.

#### Parameter values

Parameter *b *is a background swelling coefficient, i.e. it represents the swelling rate which is induced by the starting stimulus (here addition of Ca^2+^). In contrast to *a*, which reflects a positive feedback within the swelling process, it shows no clear correlation with the amount of added Ca^2+ ^over a wide range (see also Discussion). Therefore *b *is assumed to be constant in the following. In this way we can decrease the number of the necessary parameters. The error of the approximation hardly changed at all after fixing *b *to *b *= 0.021, the mean of all *b *but those of the two highest Ca^2+^concentrations (see Table 1). Simulation showed that the general outcome is not sensitive to slight variations of *b *(data not shown).

The use of fixed *b *was further supported by resulting in more consistent values of *a*, *k *and *τ *as indicated by minimized least square errors on their regression curves (see below).

Parameter *k *represents the mean volume of mitochondria during their swelling process. Higher values of *k *thus indicate a faster volume increase in the beginning of the swelling process compared to the end, e.g. a less convex/more concave swelling curve of single mitochondria, lower values may even indicate a transient shrinking (see below). With fixed *b *the parameter *k *shows a very slow exponential increase with increasing Ca^2+^-concentration (data in Table 1, figure not shown). We obtained the formula *k *= 0.692 + 0.0137 * *exp*(0.0218 1/*μ*M·[Ca]) with a relative least squared error of 0.0027.

**Figure 5:**
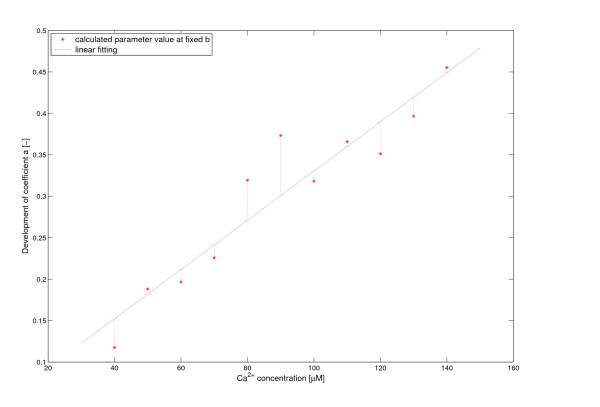
**Dependance of rate parameter *a *on the added Ca^2+^**. Linear regression formula for parameter *a *for varying Ca^2+^-concentrations: *a *= 0.00297 1/*μM*·[Ca] + 0.0333. The relative least squared error is 0.1775. The value of parameter *b *is fixed at *b *= 0.021.

**Figure 6:**
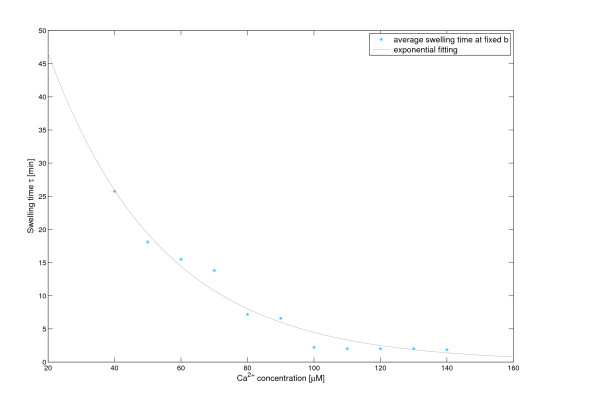
**Dependance of swelling time *τ *on the added Ca^2+^**. Linear regression formula for parameter *τ *for varying Ca^2+^-concentrations: *τ *= 83.7 min·exp(-0.0293 1/*μ*M·[Ca]). The relative least squared error is 1.7097. The value of parameter *b *is fixed at *b *= 0.021.

Whereas with increasing Ca^2+ ^*k *shifted only weakly and thus might not be overinterpreted, a more significant, probably linear correlation of Ca^2+ ^with *a *exists (see Figure 5, *a *= 0.00297 1/*μ*M·[Ca] + 0.0333, relative least squared error: 0.1775). The delay term *τ *decreases exponentially with Ca^2+ ^(see Figure 6, *τ *= 83.7 min·exp(-0.0293 1/*μ*M·[Ca]), relative least squared error: 1.7097) (for explanation of these parameters see Discussion).

Summarized, our model proposes two aspects of swelling induced by Ca^2+^: a widely Ca^2+^-independent part, describing a first order (linear) swelling process (parameter *b*) and a second part, reflecting the positive feedback loop (parameter *a*).

#### Initial shrinking of mitochondria

**Figure 7:**
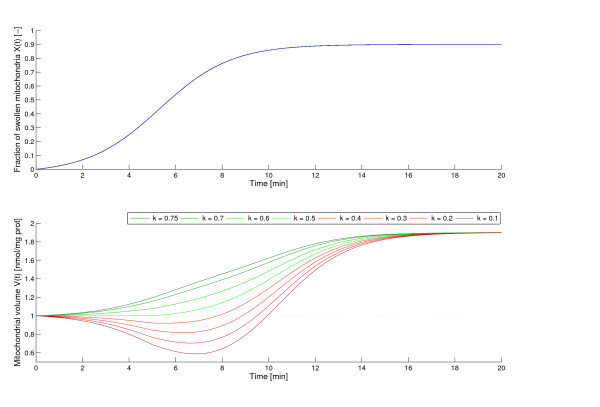
**The effect of *k *on the shape of the volume curve**. Fraction of swollen mitochondria *X*(*t*) and volume development *V *(*t*) from the model corresponding to different parameters *k*. Parameters: *a *= 0.7, *b *= 0.02, *τ *= 5.

As shown for example in [20,7,17] and [25] at high Ca^2+^-concentrations, mitochondria may go through a short phase of initial shrinking before the actual swelling begins. A possible explanation for this mitochondrial contraction may be a calcium uptake induced decrease in the transmembrane potential causing an increased respiration activity and a concomitant alteration of the mitochondrial structure. Our model shows this phenomenon with the changing of parameter *k*, the average volume of the swelling mitochondria. Figure 7 shows volume curves for different values of *k*. Obviously, a value of *k *with *kV*_*p *_<*V*_0 _means that the mitochondria first shrink, thus loose from the initial volume *V*_0_, then a fast swelling follows to reach the final volume *V*_*p*_. In other words, in mitochondria induced to volume change (here incorrectly called "swelling mitochondria") shrinking dominates swelling. It might happen e.g. by a longer duration of the shrunk stage, followed by a brief actual swelling, or by a fast and large volume drop in the beginning followed by a jump back to the swollen volume. Thus, in average the volume of the transforming mitochondria is smaller than that of the unswollen. Please note that a *kV*_*p *_>*V*_0 _does not exclude an initial shrinking, but indicates a domination of partly swollen mitochondria over shrunk at a certain time. Our model is (to our knowledge) the first which allows for the in reality observed, although mechanistically not yet fully understood initial shrinking.

#### Other organs, different inducers

**Figure 8:**
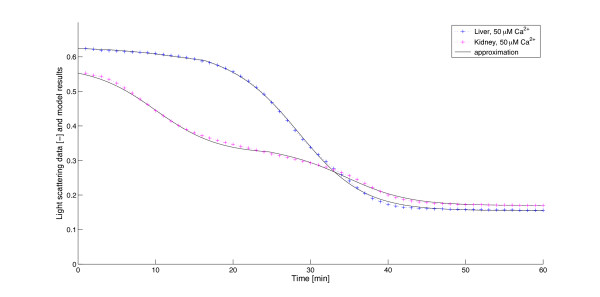
**Comparison of swelling of liver and kidney mitochondria**. The blue + signs form the swelling curve from the swelling experiment with 50 *μ*M Ca^2+ ^in liver, the red + signs with the same amount in kidney. The solid black lines are the corresponding rescaled numerical approximations of the volume (*V *(*t*)) obtained from the model. Optimized parameter values: for liver *τ *= 16.2803, *a *= 0.2544, *b *= 0.0118, *k *= 0.7360; for kidney: *τ *= 24.6519, *a *= 0.2522, *b *= 0.0193, *k *= 0.8859. Relative least squared error 7.8132e-04 for liver and 9.0954e-04 for kidney.

We tested the model with other organs and inducers. Figure 8 presents the comparison of the swelling of liver and kidney mitochondria due to the same strength of Ca^2+^-induction along with the optimal parameter values. As can be seen, the approximation by the model produces very accurate results, although the swelling curves are quite different. The difference results from differing *τ *and *k *values, while the optimal values for *a *and *b *are almost identical, which refers to differing delay/Ca^2+^-takeup time but similar feedback mechanism in the two organs.

**Figure 9:**
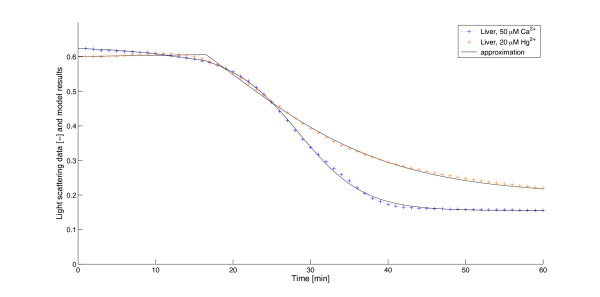
**Comparison of swelling induced by Ca^2+ ^and Hg^2+ ^in liver mitochondria**. The blue + signs form the swelling curve from the swelling experiment with 50 *μ*M Ca^2+ ^in liver, the red + signs with 20 *μ*M Hg^2+ ^in liver. The solid black lines are the corresponding rescaled numerical approximations of the volume (*V *(*t*)). Optimized parameter values: for Ca^2+ ^*τ *= 16.2803, *a *= 0.2544, *b *= 0.0118, *k *= 0.7360; for Hg^2+ ^*τ *= 16.5391, *a *= 0.0546, *b *= 0.0410, *k *= 0.6995. Relative least squared error 7.8132e-04 for Ca^2+ ^and 1.0289e-03 for Hg^2+^.

Figure 9 shows the comparison of the swelling of liver mitochondria due to Ca^2+ ^and due to Hg^2+ ^induction with the optimal parameter values. Here all the parameter values are almost identical, except for the feedback rate *a*, which is close to 0 in case of Hg^2+^. Thus, in contrast to Ca^2+^, Hg^2+^-induced swelling does not involve a feedback effect.

Clearly, further studies have to substantiate these preliminary results.

## Discussion

Although the mitochondrial permeability transition is an important biochemical process [3], the details of which are not yet fully understood, the experimentally observed swelling process can be completely displayed by the present model, including a time delay (which probably mainly reflects a signal transfer process), a second order kinetics (positive feedback) and three classes of mitochondria (unswollen, swelling, swollen) as well. The model is mathematically straightforward, yet explains the swelling process throughout the whole range more accurately than other models. The parameters of the model change in a well-determined way, consistent with the corresponding Ca^2+^-concentrations.

The introduction of second order kinetics and the delay term are major features of the model. The former one is reflected in parameter *a *and can be interpreted as follows.

At the end of the swelling process, once the pores irreversibly open, not only the formerly uptaken Ca^2+ ^gets re-released, but also the natural Ca^2+^-depot within the mitochondria, resulting in an additional increase of external Ca^2+^-concentration and thus a faster swelling of other mitochondria. This starts a chain-reaction of swelling among mitochondria. Importantly, such a feedback mechanism was shown to occur *in vivo *[26-28]. It constitutes probably at least the main base of the found positive feedback loop, and Parameter *a *describes the strength of such a positive feedback loop. This interpretation is supported by the positive relation between *a *and the starting concentration of Ca^2+ ^(*a *= 0.00297 1/*μ*M·[Ca] + 0.0333). A further proof of this is the slower swelling with insignificant parameter value *a *experienced with another inducer, Hg^2+ ^(see Figure 9).

As mentioned in the Results section, the delay term *τ *decreases exponentially with the amount of calcium. This behaviour probably is governed by signal transfer processes: a high amount of extramitochondrial Ca^2+ ^results in a faster arrival of amounts of Ca^2+ ^in the mitochondria (via the Ca^2+^-uniporter) and thus a faster binding of Ca^2+ ^to the factors inducing the mitochondrial permeability transition. The quantitative and qualitative dependency on exogenous Ca^2+ ^can be expected to vary between different types of mitochondria, e.g. in depending on number and properties of transmembrane Ca^2+^-transporter, the Ca^2+^-buffer properties of the matrix and Ca^2+^-binding places at the pores. Figure 8 shows how the different sensitivity of liver and kidney mitochondria with respect to Ca^2+ ^can be accounted for by differing *τ *and *k *values. Currently, it cannot be decided whether *τ *decreases because all mitochondria swell faster with higher Ca^2+^-concentrations, or only a (more sensitive) subpopulation starts earlier swelling.

**Figure 10:**
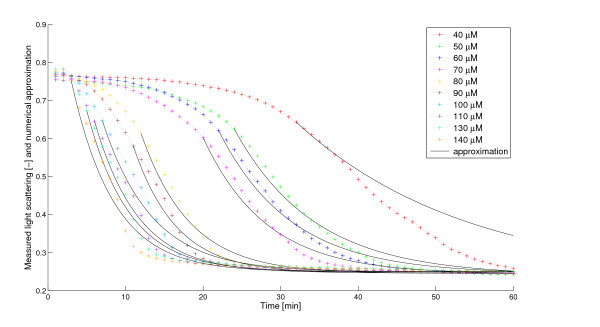
**Experimental data and simulated swelling curves with the assumption of linear kinetics (*a *= 0) and no delay (*τ *= 0)**. The color lines with the + signs are the swelling curves from the experiments at different Ca^2+^-concentrations (40 - 140 *μ*M). The solid black lines are the corresponding rescaled numerical approximations of the volume (*V *(*t*)) obtained from the model assuming linear kinetics and no delay. Parameter *b *and the starting points of the approximations were optimized.

**Figure 11:**
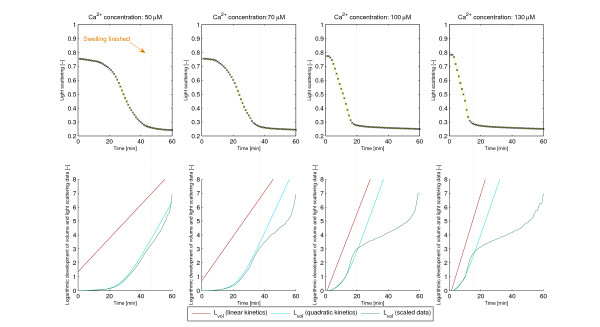
**Development of the logarithmic volume according to the experiments and simulations**. , where *V*_0 _is the initial volume before swelling, *V*_*p *_the final volume after swelling, and *V *(*t*) the volume at time *t*. Green line obtained from the experiments, red line from the model assuming linear kinetics, blue line from the model assuming quadratic kinetics.

The importance and advantage of second order kinetics and delay become obvious if we compare the model with the one that lacks these. By removing these terms (setting *a *= 0 and *τ *= 0) the intermediate volume *V*_2 _disappears and we get back the model described in [15]. Figure 10 shows the best fit of these simulations to the experimental data (cf. Figure 4). Another sign of the advantage of the new terms is the significantly better correspondence with the logarithmic volume mentioned in the Backgrounds section (see Figure 11). Obviously, the dynamics of swelling is, despite the complexity of its regulation, a rather simple, almost one-phase process, which is, once started, governed by the positive feedback via Ca^2+^-release. Only at very high (> 140 *μ*M) Ca^2+^-concentrations, the delay seems to vanish (data not shown). However, this probably is caused by experimental limitations. At high Ca^2+^-concentration the reaction rate is higher. Due to technical restrictions, some data are lost until the actual measurements start, thus omitting the delay. Here one can get a very good estimate assuming that there is no delay at all, which means *τ *= 0. In this marginal case our volume formula (5) agrees with the one in [15], thereby confirming the statement in [16], that it gives a good approximation for saturating Ca^2+^-levels. However, the transport processes naturally will need some time, even at high Ca^2+^-loads, thus the total lack of a delay does not reflect the real situation.

We also noticed that at very high external Ca^2+^-concentration, if we did not keep the value of the parameter *b *fixed, but rather let it vary freely in the optimization, then the values of *b *shifted higher (accompanied by a slightly sharper break in the curves, as expected). This may indicate the possible onset of an additional process in dynamics of swelling, possibly an additional positive feedback at high Ca^2+^-concentration.

It occurred to us that there are certain limitations in our model. As mentioned above, since we take the average of the volume over the whole mitochondrial population, currently the model cannot distinguish between subpopulations with different swelling behavior. An attempt to represent this phenomenon with a different model with an integral formula has been made in (Eisenhofer S, Hense BA, Tookos F, Zischka H: Modeling the volume change in mitochondria, submitted). Also, because of the rescaling of the optical density data, possible differences in the levels of the final optical density values after swelling at present cannot be explained (as seen in Figure 9 or comparing data from different animals). This issue needs to be addressed in further studies, especially regarding various inducers. Due to its nature the model cannot explain the exact biological mechanism of the feedback process, nevertheless the comparison with other inducers/inhibitors sheds light on some of its characteristics. Such a comparison also allows to quantify the differences in the swelling. This is of vital importance because depending on the inducer the extent and duration of permeability transition decides whether a cell dies, and if so, whether by apoptosis or necrosis [29]. Therefore the parameters have to be determined and compared in a mathematically exact way, which goes beyond a mere experimental comparison.

## Conclusions

A mathematical model of the MPT swelling process was developed, which accurately corresponds with the experimental data of the full swelling process. The model unveils a positive feedback behaviour of the swelling process and enables a quantitative understanding of the swelling process itself. It will be applied in studies of mitochondria which deviate in behaviour from the Ca^2+^-induced swelling. The application of the model to such data with the necessary change of parameter values will shed light on the biological properties of the mitochondria that are causative for these differences. Shifts of *a *for example would indicate differences concerning the positive feedback, possibly due to other amounts of Ca^2+ ^stored in the mitochondria. As the model is general in the sense that it is not designed for an induction by Ca^2+ ^only, it allows to quantitatively analyze and compare effects of other swelling inducers or inhibitors as well.

A partial differential equation has also been created based on equation (1). This reaction-diffusion equation will give more insight into the spacial dynamics, making it possible to analyze the positive feedback and the local changes of the Ca^2+^-concentration.

## Competing interests

The authors declare that they have no competing interests.

## Authors' contributions

HZ and SS performed the experiments. SE and FT designed the mathematical model. SE did the computer simulations. HZ, BH and FF helped coordinating the work and analyzing the model. BH and FT prepared the first draft of the manuscript. All authors read and approved the final version of the manuscript.
